# A novel role for protein tyrosine phosphatase 1B as a positive regulator of neuroinflammation

**DOI:** 10.1186/s12974-016-0545-3

**Published:** 2016-04-19

**Authors:** Gyun Jee Song, Myungsu Jung, Jong-Heon Kim, Hana Park, Md Habibur Rahman, Sheng Zhang, Zhong-Yin Zhang, Dong Ho Park, Hyun Kook, In-Kyu Lee, Kyoungho Suk

**Affiliations:** Department of Pharmacology, Brain Science and Engineering Institute, BK21 Plus KNU Biomedical Convergence Program, Kyungpook National University School of Medicine, Daegu, Republic of Korea; Department of Medicinal Chemistry and Molecular Pharmacology, Purdue University, West Lafayette, IN 47907 USA; Department of Ophthalmology, Kyungpook National University School of Medicine, Daegu, Republic of Korea; Department of Pharmacology, Chonnam National University Medical School, Gwangju, Republic of Korea; Department of Internal Medicine, Division of Endocrinology and Metabolism, Kyungpook National University School of Medicine, Daegu, Republic of Korea

**Keywords:** Neuroinflammation, PTP1B, Microglia, Proinflammatory cytokines, Lipopolysaccharide, Src

## Abstract

**Background:**

Protein tyrosine phosphatase 1B (PTP1B) is a member of the non-transmembrane phosphotyrosine phosphatase family. Recently, PTP1B has been proposed to be a novel target of anti-cancer and anti-diabetic drugs. However, the role of PTP1B in the central nervous system is not clearly understood. Therefore, in this study, we sought to define PTP1B’s role in brain inflammation.

**Methods:**

PTP1B messenger RNA (mRNA) and protein expression levels were examined in mouse brain and microglial cells after LPS treatment using RT-PCR and western blotting. Pharmacological inhibitors of PTP1B, NF-κB, and Src kinase were used to analyze these signal transduction pathways in microglia. A Griess reaction protocol was used to determine nitric oxide (NO) concentrations in primary microglia cultures and microglial cell lines. Proinflammatory cytokine production was measured by RT-PCR. Western blotting was used to assess Src phosphorylation levels. Immunostaining for Iba-1 was used to determine microglial activation in the mouse brain.

**Results:**

PTP1B expression levels were significantly increased in the brain 24 h after LPS injection, suggesting a functional role for PTP1B in brain inflammation. Microglial cells overexpressing PTP1B exhibited an enhanced production of NO and gene expression levels of TNF-α, iNOS, and IL-6 following LPS exposure, suggesting that PTP1B potentiates the microglial proinflammatory response. To confirm the role of PTP1B in neuroinflammation, we employed a highly potent and selective inhibitor of PTP1B (PTP1Bi). In LPS- or TNF-α-stimulated microglial cells, in vitro blockade of PTP1B activity using PTP1Bi markedly attenuated NO production. PTP1Bi also suppressed the expression levels of iNOS, COX-2, TNF-α, and IL-1β. PTP1B activated Src by dephosphorylating the Src protein at a negative regulatory site. PTP1B-mediated Src activation led to an enhanced proinflammatory response in the microglial cells. An intracerebroventricular injection of PTP1Bi significantly attenuated microglial activation in the hippocampus and cortex of LPS-injected mice compared to vehicle-injected mice. The gene expression levels of proinflammatory cytokines were also significantly suppressed in the brain by a PTP1Bi injection. Together, these data suggest that PTP1Bi has an anti-inflammatory effect in a mouse model of neuroinflammation.

**Conclusions:**

This study demonstrates that PTP1B is an important positive regulator of neuroinflammation and is a promising therapeutic target for neuroinflammatory and neurodegenerative diseases.

**Electronic supplementary material:**

The online version of this article (doi:10.1186/s12974-016-0545-3) contains supplementary material, which is available to authorized users.

## Background

Neuroinflammation, an innate immunological response of the nervous system [1], is strongly associated with many neurodegenerative diseases including Alzheimer’s disease, Parkinson’s disease, and multiple sclerosis. Therefore, neuroinflammation is now considered to be a hallmark of neurodegenerative diseases [2]. The neuroinflammatory response includes the activation of the brain’s resident innate immune cells (microglia) and macrophage infiltration and the release of inflammatory mediators, such as nitric oxide (NO), cytokines, and chemokines, which often lead to neuronal death [2]. The inflammatory activation of the microglia is considered to be an important pathological mechanism underlying the progression of neurodegenerative diseases. Therefore, tight control of microglial activation is essential for maintaining brain homoeostasis and preventing neuroinflammatory diseases.

Many pathophysiological conditions, including inflammation, diabetes, and cancer, are regulated by a delicate balance between protein tyrosine kinases and phosphatases. Protein tyrosine phosphatase 1B (PTP1B, also known as PTPN1) is a major negative regulator of the insulin and leptin signaling pathways [3–5]. Studies from mice with the *PTP1B* gene deletion have demonstrated that PTP1B is a key regulator of insulin sensitivity. Accordingly, inhibition of PTP1B is protective against diabetes [5]. Many studies on PTP1B have also been carried out in the cancer field. PTP1B expression is highly upregulated in colon and breast cancers; targeting PTP1B by genetic deletion or by pharmacological inhibitor has resulted in a better prognostic outcome [6, 7]. Neuron-specific PTP1B (−/−) mice have reduced body weight and adiposity and are hypersensitive to leptin. Therefore, PTP1B may be a negative regulator of central nervous system (CNS) leptin and insulin signaling pathways via the dephosphorylation of the Tub protein [8]. In mice fed a high-fat diet, leptin- and insulin-induced tyrosine phosphorylation of the Tub protein was reduced in the hypothalamus, which was then reversed by an intracerebroventricular (i.c.v.) administration of PTP1B anti-sense oligonucleotides. Therefore, it is thought that there are therapeutic implications for chemical inhibitors of PTP1B for patients with diabetes, obesity, and cancer [9–12].

PTP1B expression is increased under inflammatory conditions. TNF-α, a key proinflammatory cytokine, positively regulates PTP1B expression in adipocyte, hepatocyte cell lines, and mouse hypothalamus as well as in an animal model of high-fat diet-mediated obesity [13, 14]. Zabolotny et al. have reported that the p65 subunit of NF-κB binds to the PTP1B promoter in diet-induced obese mice, suggesting that PTP1B could be a target of anti-inflammatory therapies [13]. PTP1B has also been reported to be a negative regulator of IL-4-induced anti-inflammatory signaling [15]. IL-4 increased PTP1B levels and an overexpression of PTP1B suppressed IL-4-induced STAT6 signaling through a negative feedback loop in vitro. More recently, PTP1B deficiency ameliorated colitis in a dextran sulfate sodium-induced experimental model through the expansion of CD11b(+)Gr-1(+) myeloid-derived suppressor cells [16]. In contrast, there are several reports demonstrating the anti-inflammatory effect of PTP1B in macrophages [17–19]. For example, PTP1B deficiency amplified the effects of proinflammatory stimuli in macrophages. Although PTP1B is an important regulator in the inflammatory signaling pathway, it is unclear whether PTP1B contributes to the neuroinflammatory response. In the present study, we investigated the role of PTP1B in neuroinflammation using in vitro and in vivo models. We found that PTP1B expression in microglia was enhanced by LPS treatment, and the PTP1B overexpression potentiated an LPS-induced proinflammatory activation of microglia. PTP1B inhibition with a pharmacological inhibitor attenuated microglial activation in the mouse brain. This newly identified role of PTP1B may provide a novel strategy to control neuroinflammation.

## Methods

### Cell culture

An immortalized murine microglial cell line, BV-2 cells [20], was maintained in Dulbecco’s modified Eagle’s medium (DMEM) containing 5 % heat-inactivated fetal bovine serum (FBS) and 50 mg/ml gentamicin at 37 °C. A highly aggressively proliferating immortalized (HAPI) rat microglial cell line [21] and mouse primary microglial cells were maintained in DMEM containing 10 % heat-inactivated FBS, 10 U/ml penicillin, and 10 mg/ml streptomycin (Gibco) at 37 °C in a humidified atmosphere with 5 % CO_2_. All animals and experimental procedures were approved by the Institutional Review Board of Kyungpook National University School of Medicine and were carried out in accordance with the guidelines in the NIH Guide for the Care and Use of Laboratory Animals. The animals were maintained under temperature- and humidity-controlled conditions with a 12-h light/12-h dark cycle. The mouse primary microglial cultures were prepared by mild trypsinization, as previously described with minor modifications [22]. In brief, the forebrains of 3–5-day-old C57BL/6 mice were chopped and dissociated by mechanical disruption using a nylon mesh. The cells were seeded into poly-l-lysine-coated flasks. After in vitro culture for 10–14 days, the microglial cells were isolated from the mixed glial cultures by mild trypsinization. The mixed glial cultures were then incubated with a trypsin solution (0.25 % trypsin, 1 mM EDTA in Hank’s balanced salt solution) diluted 1:4 in phosphate-buffered saline (PBS; 150 mM NaCl, 5 mM phosphate, pH 7.4) containing 1 mM CaCl_2_ for 30–60 min. This resulted in the detachment of an upper layer of astrocytes; the microglia remained attached to the bottom of the culture flask. The detached layer of astrocytes was aspirated, and the remaining microglia were used for experiments. The purity of the cultures was greater than 95 %, as determined by immunocytochemistry using a rabbit polyclonal anti-Iba-1 antibody (1:1000 dilution; Wako).

### Cell transfection

BV-2 cells were transfected with HA-PTP1B using Lipofectamine™ 2000 (Invitrogen), according to the manufacturer’s instructions. The HA-tagged full-length human PTP1B complementary DNA (cDNA) in pJ3H expression vector (HA-PTP1B) was used for transfection together with an EGFP plasmid (Clontech) encoding a neomycin-resistance gene [23]. The cells were selected in the presence of 400 μg/ml G418 (Sigma) to make a cell line stably expressing HA-PTP1B. Control stable cells were made by transfecting only the EGFP plasmid. To knockdown PTP1B expression, BV-2 cells were transfected with small interfering RNAs (siRNAs) using Lipofectamine™ 2000. The cells were used for the treatments 48 h after the transfection. The PTP1B siRNA and control siRNA were purchased from Genolution Pharmaceuticals (Seoul, Korea); siCont—5′ CCUCGUGCCGUUCCAUCAGGUAGUU 3′, siPTP1B—5′ UGACCAUAGUCGGAUUAAAUU 3′.

### Measurement of nitric oxide production

The production of nitric oxide (NO) was estimated by measuring the amount of nitrite, a stable metabolite of NO. The cells were treated with lipopolysaccharide (LPS from *E. coli* 055: B5; Sigma) in the presence or absence of the inhibitors (PTP1Bi [24, 25], PP2 (Src inhibitor; Sigma), CinnGel (PTP1B inhibitor; Santa Cruz) or PDTC (NF-κB inhibitor; Sigma)). At the end of a 24-h incubation period, 50 μl of the cell culture media was mixed with an equal volume of a Griess reagent (0.1 % naphthylethylenediamine dihydrochloride and 1 % sulfanilamide in 5 % phosphoric acid) in a 96-well microtiter plate. The light absorbance was read at 540 nm and sodium nitrite was used for a standard curve.

### Assessment of cell viability

The cell viability was assessed by a modified 3-(4,5 dimethylthiazol-2-yl)-2, 5-diphenyltetrazolium bromide (MTT) assay, as previously described [26]. After LPS treatment for 24 h, either in the presence or absence of pharmacological inhibitors, the culture media was aspirated. MTT (0.5 mg/ml in PBS) was added to cells, which were then incubated at 37 °C for 4 h. The resulting formazan crystals were dissolved in DMSO. The absorbance was determined at 570 nm using a microplate reader.

### ELISA for TNF-α

BV-2 cells were treated with LPS in the presence or absence of PTP1Bi. After a 24-h incubation, the TNF-α levels in the culture media were measured using a rat monoclonal anti-mouse TNF-α antibody as the capture antibody and a goat biotinylated polyclonal anti-mouse TNF-α antibody as the detection antibody (ELISA development reagent; R&D systems), as previously described [27]. The recombinant TNF-α protein was used as a standard.

### Traditional and real-time RT-PCR

Total RNA was extracted from brain tissues or microglial cells using the TRIZOL reagent (Invitrogen), according to the manufacturer’s protocol. Reverse transcription (RT) was conducted using the Superscript II reverse transcriptase (Invitrogen) and oligo(dT) primer. The traditional PCR amplification was carried out using specific primer sets at an annealing temperature of 55–60 °C for 20–30 cycles. The PCR was performed using a C1000 Touch Thermal Cycler (Bio-Rad). For the PCR product analysis, 10 μl of each PCR reaction was electrophoresed on a 1 % agarose gel and detected under ultraviolet light following ethidium bromide staining. Glyceraldehyde 3-phosphate dehydrogenase (GAPDH) or β-actin was used as an internal control. The real-time PCR was performed using the Perfect real-time One Step SYBR PrimeScript RT-PCR Kit (Takara Bio), according to the manufacturer’s instructions, followed by detection using the ABI Prism 7000 Sequence Detection System (Applied Biosystems). GAPDH was used as an internal control. The nucleotide sequences of the primers for TNF-α, IL-1β, iNOS, GAPDH, IL-6, and β-actin were described previously [28]. For PTP1B, TC-PTP and PTP-Meg2, the primer sequences were designed based on published cDNA sequences and are as follows: PTP1B, 5′-AAGACCCATCTTCCGTGGAC-3′, 5′-ACAGACGCCTGAGCACTTTG-3′; TC-PTP, 5′-GCTGGCAGCCGTTATACTTG-3′, 5′-TGGCCAGGTGGTATAATGGA-3′; PTP-Meg2, 5′-CCTGGAATGTGGCTGTCAAG-3′, 5′- ATGCTCCCTTCAGCAGGTTT-3′.

### Western blot analysis

After various treatments, the cells were washed with PBS and lysed with RIPA lysis buffer (50 mM Tris-HCl (pH 8.0), 150 mM NaCl, 0.02 % sodium azide, 0.1 % sodium dodecyl sulfate (SDS), 1 % Nonidet P-40, 1 mM phenylmethylsulfonyl fluoride (PMSF)). Equal amounts of protein from the different treatment groups were separated by SDS-polyacrylamide gel electrophoresis (10 % gel) and transferred to nitrocellulose membranes (Amersham Biosciences). The membranes were blocked with 4 % skim milk in Tris-buffered saline Tween-20 (TBST) and then incubated with primary antibodies (goat anti-PTP1B (1:500 dilution, Santa Cruz Biotechnology), polyclonal rabbit anti-phospho- or total forms of Src (1:1000 dilution, Cell signaling), rabbit anti-IκB (1:1000 dilution, Santa Cruz), mouse anti-β-actin (1:2000 dilution, Sigma-Aldrich), mouse anti-α-tubulin (1:5000 dilution, Sigma-Aldrich), and goat anti-LCN2 (1:500 dilution, R&D systems)). After thorough washing with TBST, horseradish peroxidase-conjugated secondary antibodies were applied. The blots were developed using an enhanced chemiluminescence detection kit (SuperSignal™ West Femto, ThermoFisher).

### Immunostaining

The cells were plated and cultured on coverslips and then fixed in 4 % paraformaldehyde after indicated stimulation. Nonspecific binding sites were blocked with 5 % goat serum for 1 h at room temperature to minimize background staining. The cells were incubated overnight with the appropriate primary antibodies (goat anti-PTP1B, 1:500, Santa Cruz; rabbit anti-Iba-1, 1:1000, Wako) at 4 °C. The cells were washed and incubated with secondary antibodies (anti-goat FITC, 1:500 and anti-rabbit Cy3, 1:500; Jackson ImmunoResearch) and DAPI (for nuclei staining). For histochemical analysis, the mice were transcardially perfused with saline and whole brains were fixed in 4 % paraformaldehyde for 72 h. The fixed brains were incubated in 30 % sucrose for 72 h and embedded in Optimal Cutting Temperature (O.C.T.) compound (Tissue-Tek) and then cut into 12-μm-thick sagittal sections. The sections were permeabilized with 0.3 % Triton X-100 and blocked with 1 % BSA and 5 % normal donkey serum for 1 h at room temperature. The brain sections were incubated with primary antibodies (goat anti-PTP1B (1:500 dilution) and rabbit polyclonal anti-Iba-1 (1:500 dilution)) at 4 °C overnight, followed by an incubation for 1 h at room temperature with secondary antibodies (Cy3-conjugated donkey anti-rabbit IgG, FITC-conjugated donkey anti-goat IgG; Jackson ImmunoResearch Laboratories). The anti-fade mounting medium containing DAPI (VECTASHIELD, Vector laboratories) was used for mounting and counterstaining. Tiled images of each section were captured with a CCD color video camera (Olympus D70) through a ×63 objective lens attached to a microscope (Olympus BX51).

### Mouse model of neuroinflammation

LPS was administered intraperitoneally (i.p.) to induce neuroinflammation in mice, as previously described [28]. All experiments were carried out on 9–11-week-old male C57BL/6 mice (25–30 g) supplied by Koatech (Pyongtaec, Korea). To evaluate the expression of PTP1B in the brain under inflammatory condition, LPS was injected i.p. at a dose of 5 mg/kg. The brains were collected 6, 24, or 48 h after LPS administration. To assess the effect of PTP1Bi on neuroinflammation, animals were divided into four experimental groups: group 1, no reagents or treatment; group 2, treated with PTP1Bi; group 3, treated with LPS and PTP1Bi; and group 4, treated with LPS and 0.5 % DMSO diluted in saline containing 5 % propylene glycol. PTP1Bi was diluted in saline containing 5 % propylene glycol. DMSO was included in the vehicle because PTP1Bi was dissolved in DMSO. LPS (5 mg/kg) was administered i.p. for a single challenge. PTP1Bi or vehicle was administered intracerebroventricularly (i.c.v.). For histological analysis, the mice were anesthetized 48 h after the LPS injection and then transcardially perfused with saline and then with 4 % paraformaldehyde. Microglial activation was assessed by Iba-1 staining. For mRNA and protein analysis, the mice were anesthetized and transcardially perfused with saline. The brains were removed and stored at −80 °C until analysis. At least three animals were used for each experimental group. Immunohistological intensity analysis of Iba-1 staining was performed using Image J software (NIH, Bethesda, MD, USA) as previously described [29]. The image was set with a binary threshold of 50 % of the background level, and then the particles were converted to a subthreshold image area with a size of 20 to 300 pixels, which was judged as showing the Iba-1-positive cells. This range (20 to 300 pixels) was obtained from the analyzed size of Iba-1-positive cells from six sections for each animal. To count the Iba-1 positive cells, five squares (300 × 300 μm) were placed around the injection site in the subthreshold image of the six independent sections, and the cells in the five squares were counted and statistically analyzed.

### Statistical analysis

All data are presented as mean ± SE from three or more independent experiments, unless stated otherwise. The statistical comparisons between the different treatments were made either by a Student’s *t* test or by a one-way ANOVA, using the GraphPad PRISM and Excel. To determine the statistical significance of more than two groups, the values were compared using a one-way ANOVA followed by a Tukey’s multiple comparison test (parametric test) or a one-way ANOVA with a Dunn’s test (non-parametric test). For the comparison of three groups, the unpaired two-tailed Student’s *t* test was used, followed by a Mann-Whitney correction for the non-parametric data.

## Results

### PTP1B expression in the mouse brain is increased after LPS injection

**Fig. 1:**
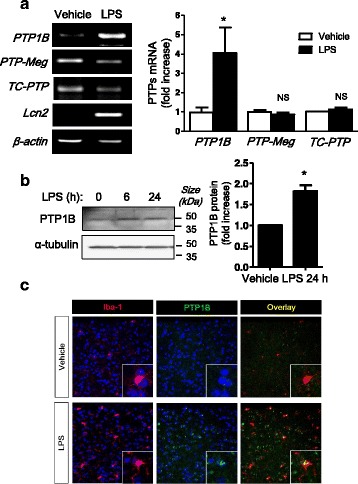
PTP1B expression is upregulated by LPS in the mouse brain. **a** mRNA expression of PTP1B in the brain 24 h after LPS injection (5 mg/kg). The expression of PTP-Meg2 and TC-PTP were also measured by RT-PCR along with the inflammatory maker, Lcn2. The band intensity of PTP1B, PTP-Meg2, and TC-PTP mRNA from three to four independent experiments was measured and normalized to β-actin expression. *p < 0.05 versus vehicle control. *NS* not significant. **b** Western blot analysis of PTP1B protein expression levels in the brain 6 h or 24 h after LPS injection. α-tubulin was used as a loading control. The graphs show the average band intensity of PTP1B and the error bars show standard error from 5 animals 24 h after LPS injection. *p < 0.05 versus vehicle-injected control, analyzed by Student’s *t* test. **c** PTP1B expression (*green*) in the cortex area. PTP1B is co-localized with Iba-1 (*red*), a microglia marker, 24 h after LPS injection (5 mg/kg). *Arrows* indicate colocalization of PTP1B and Iba-1 expression. Nuclei were stained with DAPI (*blue*)

We first investigated whether PTP1B expression is regulated by inflammatory conditions in the mouse brain. For an animal model of neuroinflammation, we used LPS-injected mice. Whole brains were collected 24 h after an i.p. injection of LPS (5 mg/kg). The gene expression levels of PTP1B, TC-PTP (also known as PTPN2, a phosphatase highly homologous to PTP1B) and PTP-Meg2 (also known as PTPN9), members of non-receptor types of PTP family, were assessed by RT-PCR using gene-specific primers. The PTP1B expression was increased after 24 h (Fig. 1a). The inflammatory marker Lcn2 was also highly upregulated by LPS. PTP-Meg2 has an effect on insulin signaling in a manner similar to PTP1B [30, 31]. TC-PTP and PTP-Meg2 expression levels were not increased by LPS, indicating a specific induction of PTP1B under inflammatory conditions. We next tested whether PTP1B protein levels were also increased by LPS. After LPS stimulation, PTP1B protein levels were modestly higher in the total brain lysates 24 h after LPS injection compared to saline-administered brain lysates (Fig. 1b). Because microglia are the resident immune cells in the CNS and participate in the initiation and propagation of an inflammatory response, we examined PTP1B expression in brain microglia using immunostaining. PTP1B protein expression was increased in the cytoplasm of Iba-1-positive microglia after LPS treatment (Fig. 1c).

### PTP1B expression in cultured microglial cells is increased by LPS

**Fig. 2:**
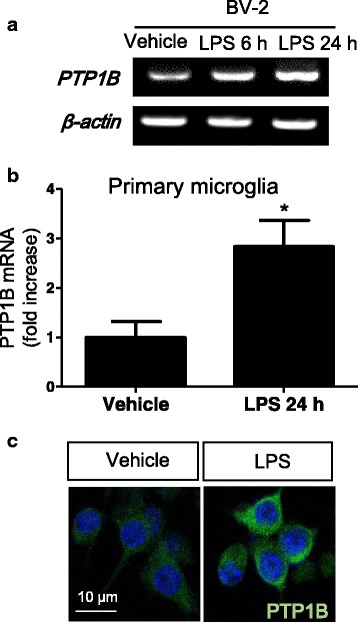
PTP1B expression is upregulated by LPS in microglia. **a** mRNA expression of PTP1B in BV-2, a mouse microglial cell line 6 and 24 h after LPS treatment (100 ng/ml). **b** PTP1B expression in primary microglia as measured by real-time RT-PCR. Data from triplicate determination are shown (mean and standard error). *p < 0.05 versus vehicle-treated control, analyzed by Student’s *t* test. **c** Immunostaining of PTP1B (*green*) in BV-2 microglial cell line with or without LPS (100 ng/ml) treatment for 24 h. Nuclei were stained with DAPI (*blue*)

Having shown LPS-induced PTP1B upregulation in mouse brain and the localization of PTP1B expression in brain microglia, we next utilized the BV-2 mouse microglial cell line to further investigate the regulation of PTP1B expression. PTP1B, but not PTP-Meg, mRNA levels were increased after 24-h stimulation with LPS (100 ng/ml) (Fig. 2a). LPS-induced PTP1B mRNA expression was similarly observed in primary microglial cultures (Fig. 2b). Immunostaining using anti-PTP1B antibody revealed a cytoplasmic expression of PTP1B protein in the BV-2 microglial cells as well as its upregulation after LPS treatment (Fig. 2c). Taken together, our results indicate that inflammatory stimuli increased PTP1B expression levels in brain microglia.

### PTP1B overexpression in LPS-stimulated microglia potentiates NO production and the expression of proinflammatory mediators

**Fig. 3:**
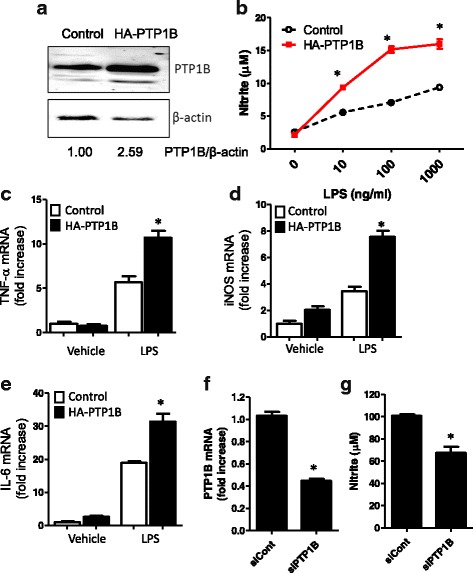
PTP1B potentiates LPS-induced NO production. **a** PTP1B expression in BV-2 cells overexpressing HA-PTP1B. Western blot analysis showed a 2.59-fold increase in PTP1B expression in cells stably expressing HA-PTP1B. β-actin was used as a loading control. **b** LPS-induced NO production in BV-2 cells with or without PTP1B overexpression. BV-2 cells were treated with LPS at the indicated concentration (0–1000 ng/ml) for 24 h. Nitrite accumulation was measured using the Griess reaction. Real-time RT-PCR was performed to determine mRNA expression of TNF-α (**c**), iNOS (**d**), and IL-6 (**e**) in either control or HA-PTP1B-transfected BV-2 cells after LPS (100 ng/ml) treatment for 6 h. *p < 0.05 versus control BV-2 with LPS; analyzed by one-way ANOVA with Tukey’s multiple comparison test. **f** PTP1B expression in BV-2 cells transfected with PTP1B siRNA (siPTP1B). Real-time RT-PCR was performed to determine mRNA expression of PTP1B. **g** LPS-induced NO production in BV-2 cells after knockdown of PTP1B expression. The data were expressed as the mean ± SEM (*n* = 3). *p < 0.05 versus control BV-2 cells transfected with control siRNA (siCont)

To investigate the functional role of increased PTP1B expression in microglia under inflammatory conditions, we established a line of BV-2 microglial cells stably overexpressing HA-PTP1B. The enhanced PTP1B protein expression in the stable HA-PTP1B transfectants was confirmed by western blot analysis (Fig. 3a). Since NO production is an indicator of microglial inflammatory activation, we investigated the effect of a forced upregulation of PTP1B on LPS-induced NO production. Parental BV-2 cells and the stable HA-PTP1B transfectants were stimulated with LPS for 24 h. Subsequently, the accumulated nitrite in the culture media was estimated using Griess reaction as an index for NO synthesis. NO production was increased by LPS in a dose-dependent manner (Fig. 3b). PTP1B overexpression potentiated LPS-induced NO production at all LPS concentrations. As increased inflammatory cytokine levels are an indicator of hyperactivated microglia [32, 33], the effects of PTP1B overexpression on the production of proinflammatory cytokines was also determined in microglial cells by RT-PCR. Indeed, PTP1B overexpression potentiated the LPS-induced expression of TNF-α, iNOS, and IL-6 mRNA (Fig. 3c–e). To knockdown PTP1B expression, BV-2 cells were transfected with siRNA against PTP1B. We obtained 60 % downregulation of PTP1B expression (Fig. 3f), and the PTP1B knocking down reduced LPS-induced NO production (Fig. 3g).

### Inhibition of PTP1B suppresses microglial inflammatory activation

**Fig. 4:**
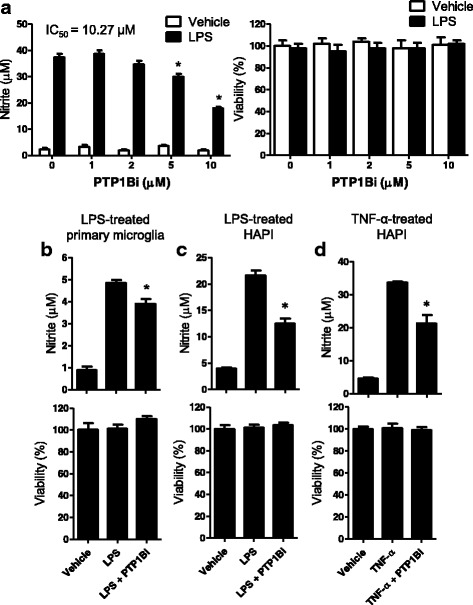
PTP1B inhibitor suppressed LPS-induced NO production in microglial cells. **a**. BV-2 microglial cells were treated with LPS (100 ng/ml) for 24 h after a 1 h pretreatment with the indicated concentration of PTP1Bi (PTP1B inhibitor). **b**. Primary microglial cells were pretreated with PTP1Bi (5 μM) and stimulated with LPS (50 ng/ml) for 24 h. **c**. HAPI cells were treated with PTP1Bi (10 μM) for 1 h and stimulated with LPS (100 ng/ml) (**c**) or TNF-α (10 ng/ml) (**d**) for 24 h. The nitrite content was measured using the Griess reaction, and PTP1Bi cytotoxicity was assessed by the MTT assay. The data were expressed as the mean ± SEM (*n* = 3). *p < 0.05 versus LPS or TNF-α only; analyzed by one-way ANOVA with Tukey’s multiple comparison test

**Fig. 5:**
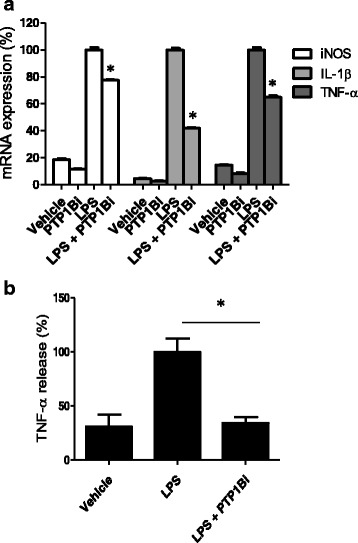
PTP1B inhibitor suppressed expression of iNOS, IL-1β, and TNF-α in LPS-stimulated BV-2 microglial cells. BV-2 microglial cells were treated with LPS (100 ng/ml) in the presence or absence of 10 μM PTP1Bi for 6 h for RT-PCR analysis (**a**) and 24 h for TNF-α ELISA analysis (**b**). After treatment, total RNA was isolated and specific mRNA levels were determined by real-time RT-PCR. Levels of iNOS, IL-1β, and TNF-α were normalized to GAPDH levels and expressed as percent value (*n* = 3). Levels of LPS-only treated cells were set to 100 %. **b**. The culture media of BV-2 cells after treatment was collected and subjected to a TNF-α sandwich ELISA. The data were expressed as the mean ± SEM and are representative of the results obtained from four independent experiments. *p < 0.05 versus LPS-only treatment; one-way ANOVA with Tukey’s multiple comparison test

PTP1B overexpression potentiated microglial production of NO and proinflammatory cytokines following LPS treatment. These results led us to hypothesize that PTP1B inhibition may inhibit microglial activation. To test this hypothesis, we used PTP1Bi, a PTP1B specific inhibitor, which we previously developed [25, 34]. PTPs share a conserved catalytic domain for the phosphatase enzyme activity. Nevertheless, PTP1B inhibitor (indicated as PTP1Bi in this study), originally called compound 2, has been shown to be highly specific for PTP1B [24, 25]. Firstly, we investigated the effect of PTP1Bi on NO production in LPS-stimulated BV-2 microglial cells. The BV-2 cells were pretreated with different concentrations of PTP1Bi before LPS stimulation. LPS-induced NO levels were decreased by PTP1Bi in a dose-dependent manner (IC_50_ value of 10.27 μM) (Fig. 4a). PTP1Bi itself did not alter the basal levels of NO production. No significant cytotoxicity was observed with PTP1Bi at the concentrations tested as determined by the MTT assay (Fig. 4a right). The inhibitory effect of PTP1Bi on NO production was also observed in mouse primary microglial cells (Fig. 4b) and in HAPI cells, a rat microglial cell line (Fig. 4c). TNF-α-induced NO production was also inhibited by PTP1Bi (Fig. 4d). Next, we examined whether PTP1Bi could also inhibit the production of proinflammatory cytokines. A pretreatment with PTP1Bi inhibited LPS-induced proinflammatory molecules, including iNOS, IL-1β, TNF-α, and COX-2 (Fig. 5a), as measured by RT-PCR. Moreover, PTP1Bi significantly inhibited LPS-induced TNF-α protein release in the microglial culture media, as measured by ELISA (Fig. 5b). We obtained similar findings with commercially available PTP1B inhibitor, CinnGel (Additional file 1: Figure S1).

### Src is a target molecule of PTP1B action in microglial activation

**Fig. 6:**
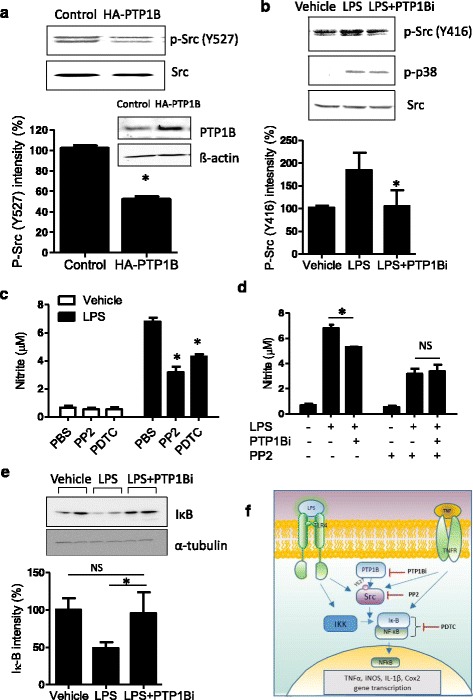
The role of PTP1B in Src-dependent microglial activation. **a** PTP1B overexpression dephosphorylated Src at Y527, a negative regulatory site. The phosphorylation of Src Y527 was quantified and normalized to total Src in either control or HA-PTP1B-transfected BV-2 cells. The graph shows the average value of phosphorylation of Y527/β-actin from four independent experiments. *p < 0.05 control versus HA-PTP1B-transfected BV-2, Student *t* test. **b** The phosphorylation of Src Y416 and p38 was quantified and normalized to total Src levels following treatment of BV-2 cells with LPS (100 ng/ml) or PTP1Bi (10 μM) for 30 min. The graph shows the average value of phosphorylation of Y416/total Src expression from four independent experiments. *p < 0.05 versus LPS only; analyzed by one-way ANOVA with Tukey’s multiple comparison test. **c** LPS (100 ng/ml)-induced nitrite production was measured in BV-2 after PP2 (Src kinase inhibitor, 5 μM) or PDTC (ammonium pyrrolidinedithiocarbamate, NF-κB inhibitor, 20 μM) treatment for 24 h. **d** BV-2 cells were pretreated with 10 μM PTP1Bi or 10 μM PP2 for 1 h and then treated with LPS for 24 h as indicated. Nitrite levels were measured by Griess solution. **e** BV-2 cells were pretreated with PTP1Bi for 1 h and then treated with LPS (100 ng/ml) for 30 min. IκB degradation by LPS was measured by western blotting. IκB intensity was measured from four independent experiments and normalized to α-tubulin. The data were expressed as the mean ± SEM (*n* = 4). *p < 0.05 versus LPS only; analyzed by one-way ANOVA with Tukey’s multiple comparison test. **f** Diagram depicting a mechanism by which PTP1B may promote proinflammatory cytokine production. PTP1B activates Src through dephosphorylation of Y527. Src may activate NF-κB and increase the production of proinflammatory molecules. *NS* not significant

We next investigated the mechanisms by which PTP1B potentiated the LPS-induced inflammatory activation of microglia. Based on literature search, we hypothesized that Src tyrosine kinase may be a potential substrate of PTP1B in microglia because Src has a negative regulatory phosphorylation site (tyrosine 527, Y527). PTP1B may dephosphorylate Src at this negative regulatory site, leading to Src kinase activation, as previously reported in breast cancer cell line [7]. This possibility was tested using the BV-2 microglial cells overexpressing PTP1B and PTP1Bi. The overexpression of PTP1B in BV-2 cells reduced Src phosphorylation at Y527 (45.7 % reduction) (Fig. 6a), consistent with previous observations in a colon cancer cell line [6]. Src activation was increased by LPS, as measured by Y416 phosphorylation (the kinase active site) of Src. This Src activity was significantly inhibited by PTP1Bi pretreatment (Fig. 6b). The PTP1Bi did not alter the levels of tyrosine phosphorylation of p38, demonstrating a specific effect of PTP1B on Src phosphorylation. Because PTP1B overexpression enhanced NO production in LPS-stimulated microglial cells (Fig. 3b), we next determined whether Src was involved in LPS-induced microglial activation. For this, BV-2 cells were treated with LPS in the presence or absence of PP2, a Src inhibitor; subsequently, NO production was measured. PP2 significantly inhibited LPS-induced NO production in microglia, to a similar extent as PDTC, an NF-κB inhibitor (Fig. 6c). The inhibition of LPS-induced NO production by PP2 pretreatment was dose-dependent (Additional file 1: Figure S2). Next, we asked whether PTP1B-mediated microglial activation was dependent on Src activity by examining the anti-inflammatory effects of PTP1Bi in microglial cells pretreated with a Src inhibitor. PP2 treatment abolished anti-inflammatory effect of PTP1Bi in microglia (Fig. 6d). These data suggest that PTP1B-mediated microglial activation is dependent on Src activity. NF-κB plays an important role in the transcriptional regulation of proinflammatory mediators. The blockade of NF-κB transcriptional activity can suppress iNOS and proinflammatory cytokines, such as IL-1β and TNF-α. We therefore investigated the effect of PTP1Bi on NF-κB activity. PTP1Bi decreased LPS-induced NF-κB activity through the suppression of IκB degradation (Fig. 6e). These data suggest that PTP1B can act as a proinflammatory factor via dephosphorylation of Src at Y527 and NF-κB activation in microglia (schematically summarized in Fig. 6f).

### The PTP1B inhibitor limits microglia-mediated neuroinflammation in vivo

**Fig. 7:**
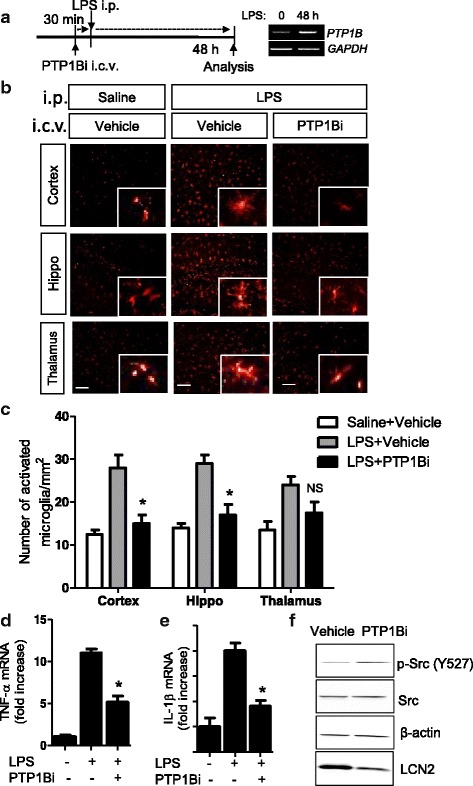
PTP1B inhibitor suppressed microglial activation in a mouse neuroinflammation model. **a** C57BL/6 mice were injected i.c.v. with vehicle (saline containing 0.5 % DMSO and 5 % propylene glycol) or PTP1B inhibitor (diluted in saline containing 5 % propylene glycol). At 30 min after the injection of PTP1B inhibitor, mice were injected i.p. with LPS (5 mg/kg). The mice were anesthetized and transcardially perfused with ice-cold saline 48 h after the LPS injection. The expression of PTP1B in the brain 48 h after the LPS injection was measured by RT-PCR. GAPDH was used for the loading control. **b** The brains were removed and the sections were stained with Iba-1 (a marker for microglia). Iba-1-positive cells were observed in the cortex, hippocampus (hippo), and thalamus region of mouse brains. *Scale bar*, 50 μm. **c**. The graph shows activated microglial cell number per square millimeter. *NS* not significant. The expression levels of proinflammatory genes were determined by real-time RT-PCR 48 h after the LPS injection. Levels of TNF-α (**d**) and IL-1β mRNA (**e**) were normalized to β-actin levels and expressed as fold increase. *p < 0.05 versus LPS + vehicle-injected animals; analyzed by one-way ANOVA with Tukey’s multiple comparison test. **f** Phosphorylation of Y527 Src in brain 48 h after LPS i.p. injection with or without PTP1Bi i.c.v. administration. Phospho (Y527)- and total Src protein levels were determined by western blot analysis. β-actin levels were used as loading controls. Lcn2 was used as a neuroinflammatory marker

Finally, we examined whether the PTP1B inhibitor limited neuroinflammation. Microglia activation is a hallmark of neuroinflammation [33, 35–38]. Therefore, the brain tissues were collected and stained with anti-Iba-1 antibody, a microglia marker, to evaluate the intensity of Iba-1 staining and microglial morphological changes 48 h after LPS i.p. injection, when the PTP1B expression in the brain remained elevated (Fig. 1a). LPS significantly increased the number of Iba-1-positive cells and hypertrophic microglia (Fig. 7b, c). Interestingly, the inhibition of PTP1B activity via PTP1Bi i.c.v. injection significantly reduced LPS-induced microglial activation 48 h after LPS injection. To confirm the anti-inflammatory effect of PTP1Bi in vivo, proinflammatory cytokine expression levels were also measured in brain tissues after LPS and PTP1Bi injection. The expression levels of TNF-α and IL-1β mRNA were significantly diminished by PTP1B inhibition in the inflammatory brain as measured by real-time RT-PCR (Fig. 7d, e). PTP1Bi injection increased Src phosphorylation at Y527, further confirming PTP1B’s effects on Src phosphorylation at Y527 in vivo (Fig. 7f). Taken together, inflammatory stimuli increased PTP1B expression to induce microglial activation in the brain. Inhibiting PTP1B activity under inflammatory conditions prevented microglial inflammatory activation in vitro and in vivo.

## Discussion

We demonstrated for the first time that the PTP1B is expressed at high levels in activated microglia and elevated PTP1B expression enhanced LPS-induced proinflammatory cytokine levels. Notably, we have shown that PTP1B overexpression caused a reduction of Src phosphorylation at the inhibitory tyrosine 527 residue, suggesting that PTP1B-mediated Src activation leads to an enhanced proinflammatory response in microglia. We further showed that i.c.v. administration of a small-molecule inhibitor of PTP1B attenuated the LPS-induced neuroinflammation in mice.

PTP1B is a major negative regulator of insulin and leptin signaling via direct dephosphorylation of the insulin receptor and leptin receptor-associated Janus kinase 2 (JAK2) [39, 40]. Mice lacking PTP1B exhibit increased insulin sensitivity and are resistant to obesity [5, 41]. PTP1B-deficient mice are protected from diet-induced obesity through modulation of energy balance, insulin sensitivity, and body fat stores [41]. Similar results were obtained after the injection of PTP1B anti-sense oligonucleotide into mice [42]. Based on these observations, PTP1B has emerged as a highly validated, attractive target for the treatment of diabetes as well as obesity.

Numerous compounds have been developed as PTP1B inhibitors and some have progressed to clinical trials for diabetes [34, 43–46]. Ertiprotafib was developed as a PTP1B inhibitor for the treatment of type 2 diabetes and progressed to a phase II clinical trial. Ertiprotafib activates the peroxisome proliferator-activated receptor (PPAR) alpha and PPAR gamma and is able to drive adipocyte differentiation [46]. Furthermore, Ertiprotafib is also a potent inhibitor of IκB kinase-beta (IKK-β), which contributes to its anti-inflammatory properties [47]. SA18 and SA32, newly developed PTP1B inhibitors, exhibited anti-obesity effects in a mouse model by suppressing weight gain. These compounds also have anti-inflammatory properties by inhibiting IKK-β, with IC_50_ values of 4.7 ± 1.0 and 14 ± 2 μM, respectively [48]. These data suggested the idea that PTP1B is a potent proinflammatory mediator, which is supported by the results of this study.

PTP1B is expressed in many tissue types including skeletal muscle, liver, adipocytes, and brain [40]. PTP1B upregulation in obese and diabetic animals and humans has been reported in many studies [49–52]. It is well accepted that obesity has an inflammatory status, including an elevation of the proinflammatory cytokines, TNF-α, IL-1β, and IL-6 in adipose tissues and sera. Zabolotny reported that TNF-α treatment induced PTP1B mRNA and protein expression in cultured adipose cells, the liver, and the arcuate nucleus of hypothalamus [13]. The mechanism of PTP1B overexpression in certain obese and diabetic states has been explained by inflammatory molecules, since TNF-α acts as a positive regulator of PTP1B [13]. NF-κB binds to the PTP1B promoter in vitro and in vivo. More recently, TrKB has been reported as a direct PTP1B substrate in the brain and the pretreatment of PTP1B inhibitor increases BDNF-induced neurite outgrowth [53]. Together, these data suggest that PTP1B overexpression is characteristic of inflammatory diseases and PTP1B inhibition could be useful anti-inflammatory and neurotrophic therapies.

Reports of the role of PTP1B in inflammation are somewhat inconsistent. Several studies have reported that PTP1B potentiated proinflammatory response. Other studies have shown that PTP1B gene ablation increases proinflammatory cytokines [17–19]. González-Rodríguez reported that PTP1B-deficiency protects against inflammation in white adipose tissue in age-associated obesity [54]. In macrophages, PTP1B negatively regulates MyD88- and TRIF-dependent proinflammatory cytokine [19], while PTP1B positively modulates palmitate-induced cytokine production in macrophages [18], IL-10-induced anti-inflammatory response in macrophages [55, 56] and LPS-induced proinflammatory cytokines in microglia (this study). Taken collectively, previous studies suggest that differential effects of PTP1B on inflammation may be dependent on (1) cell types such as microglia, adipocytes, macrophages, and liver cell line and (2) the inflammatory stimulus such as LPS-, IFN-γ-, or diet-induced obesity.

PTP1B has been reported to be the major PTP that dephosphorylates and activates Src in several breast cancer cell lines [7, 57] and colon cancer [6]. PTP1B inhibition or genetic ablation significantly enhanced the phosphorylation of the Src tyrosine 527 residue, a negative regulatory site for the Src activity [58], thus decreasing Src activity [6, 7, 59]. These studies suggest that PTP1B can act as an important activator of Src by dephosphorylation at Y527 of Src and elevated PTP1B can increase tumorigenicity by activating Src. Although the Src kinase has well-known oncogenic properties in many cancers, Src also plays an important role in inflammatory signaling (reviewed in [60, 61]). Src activation both in cancer and inflammatory cells is mainly driven by proinflammatory cytokines within the tumor microenvironment [62, 63]. Src activity mediates cytokine/chemokine production [64], underscoring the importance of Src in inflammatory signaling. The important role of Src kinase in macrophage-mediated inflammatory responses has been intensively studied (reviewed in [61]). LPS increased Src family kinase (SFK) activity in a dose- and time-dependent manner [65].

From our observation in this study, we propose that PTP1B plays a central role in producing proinflammatory cytokines in microglia through the modulation of Src activity. To confirm whether PTP1B regulates Src activity in microglia, we demonstrated that phosphorylation of the negative regulatory site (Y527) was significantly decreased by PTP1B overexpression (Fig. 6). Furthermore, LPS-induced NO production was significantly increased in cells overexpressing PTP1B and significantly inhibited by treatment of PP2, a Src inhibitor, or PTP1Bi compared to LPS-only treatment. These inflammatory processes involve closely related chemical mediators, such as NO, reactive oxygen species (ROS), prostaglandin E_2_ (PGE_2_), and various cytokines including TNF-α. Chronic inflammation is persistent inflammation characterized by tissue injury and has a longer recovery time. In vitro, Src reactivation experiments confirmed the ability of PTP1B to dephosphorylate and activate Src. As we observed in Fig. 6, PTP1B overexpression in HEK293 cells caused a twofold increase of endogenous Src activity [7]. A subset of PTPs can dephosphorylate Src family tyrosine kinases at the conserved autophosphorylation site (Y416), leading to Src inactivation, whereas PTP1B activates Src by dephosphorylation of a C-terminal inhibitory tyrosine residue, Y527 [59]. The negative regulatory C-terminal phosphorylation site Y527 in Src kinase is one of the well-known substrates of PTP1B. Phosphorylated Y527 interacts with the SH2 domain of Src, leading to the suppression of its kinase activity [66]. PTP1B can also activate Src in focal adhesions and integrin signaling [67, 68] as well as in insulin signaling [69].

In this study, we used PTP1Bi, a PTP1B inhibitor, which we previously developed [70]. Selectivity is one of the major issues in the development of PTP1B inhibitors as pharmaceutical drugs. Because all PTPs share a high degree of structural conservation in their active site, selectivity is the major road block in developing PTP1B-specific inhibitors. PTP substrate recognition requires both phosphorylated tyrosine and its adjacent flanking residues [71]. In addition, the discovery of a second aryl phosphate-binding site adjacent to the active site in PTP1B [72] has allowed us to focus on a strategy for developing bidentate PTP inhibitors that bind to both the active site and a unique adjacent peripheral site. Using this approach, we have obtained several small-molecule PTP1B inhibitors, some of which represent the most potent and selective PTP1B inhibitors reported to date [73–75]. PTP1Bi has been used in many studies to investigate the role of PTP1B [57, 76, 77].

Although the protective role of PTP1B inhibition on inflammatory conditions such as obesity-induced diabetes, colitis [16, 48], and LPS-induced neuroinflammation (Fig. 7) has been observed, the ability of PTP1B inhibitor to protect against neurodegenerative diseases including Alzheimer’s disease, Parkinson’s disease, and multiple sclerosis remains unknown. This study is the first to characterize the role of PTP1Bi in LPS-induced microglial activation and an LPS-injected neuroinflammation mouse model. Since microglial hyperactivation is a hallmark of neurodegenerative diseases, the anti-inflammatory effect of PTP1Bi would be beneficial for these diseases. In particular, PTP1B expression is upregulated by inflammatory stimuli, which increases the production of proinflammatory factors such as NO and cytokines. Thus, the inflammation-PTP1B-proinflammatory cytokine production cycle is a positive feedback loop that can contribute to chronic inflammatory conditions.

## Conclusions

Our study provides strong evidence that PTP1B expression is upregulated by inflammatory stimuli and that upregulated PTP1B promotes microglial activation and functions as a critical positive regulator of neuroinflammation. Inhibition of PTP1B activity for the regulation of inflammation provides a novel therapeutic strategy for neuroinflammatory and neurodegenerative diseases.

## Additional file

Additional file 1: This contains Figures S1 and S2. Figure S1. The PTP1B inhibitor, CinnGel, suppressed LPS-induced NO production in microglial cells. BV-2 microglial cells were treated with LPS (100 ng/ml) for 24 h after 1 h pretreatment with the indicated concentrations of CinnGel. The nitrite content was measured using the Griess reaction (a) and cytotoxicity of PTP1Bi was assessed by the MTT assay (b). The data were expressed as the mean ± SEM (*n* = 3). **p* < 0.05 versus LPS only, one-way ANOVA with Tukey’s multiple comparison test. Figure S2. The Src inhibitor, PP2, suppressed LPS-induced NO production in microglial cells. BV-2 microglial cells were treated with LPS (100 ng/ml) for 24 h after 1 h pretreatment with the indicated concentrations of PP2. The nitrite content was measured using the Griess reaction (a) and cytotoxicity of PTP1Bi was assessed by the MTT assay (b). The data were expressed as the mean ± SEM (*n* = 3). **p* < 0.05 versus LPS only, one-way ANOVA with Tukey’s multiple comparison test. (PDF 174 kb)

## References

[1] Hoogland IC, Houbolt C, van Westerloo DJ, van Gool WA, van de Beek D (2015). Systemic inflammation and microglial activation: systematic review of animal experiments. J Neuroinflammation.

[2] Frank-Cannon TC, Alto LT, McAlpine FE, Tansey MG (2009). Does neuroinflammation fan the flame in neurodegenerative diseases?. Mol Neurodegener.

[3] Tsou RC, Bence KK (2012). Central regulation of metabolism by protein tyrosine phosphatases. Front Neurosci.

[4] Yip SC, Saha S, Chernoff J (2010). PTP1B: a double agent in metabolism and oncogenesis. Trends Biochem Sci.

[5] Elchebly M, Payette P, Michaliszyn E, Cromlish W, Collins S, Loy AL, Normandin D, Cheng A, Himms-Hagen J, Chan CC (1999). Increased insulin sensitivity and obesity resistance in mice lacking the protein tyrosine phosphatase-1B gene. Science.

[6] Zhu S, Bjorge JD, Fujita DJ (2007). PTP1B contributes to the oncogenic properties of colon cancer cells through Src activation. Cancer Res.

[7] Bjorge JD, Pang A, Fujita DJ (2000). Identification of protein-tyrosine phosphatase 1B as the major tyrosine phosphatase activity capable of dephosphorylating and activating c-Src in several human breast cancer cell lines. J Biol Chem.

[8] Prada PO, Quaresma PG, Caricilli AM, Santos AC, Guadagnini D, Morari J, Weissmann L, Ropelle ER, Carvalheira JB, Velloso LA, Saad MJ (2013). Tub has a key role in insulin and leptin signaling and action in vivo in hypothalamic nuclei. Diabetes.

[9] Bakke J, Haj FG (2015). Protein-tyrosine phosphatase 1B substrates and metabolic regulation. Semin Cell Dev Biol.

[10] He RJ, Yu ZH, Zhang RY, Zhang ZY (2014). Protein tyrosine phosphatases as potential therapeutic targets. Acta Pharmacol Sin.

[11] Tamrakar AK, Maurya CK, Rai AK (2014). PTP1B inhibitors for type 2 diabetes treatment: a patent review (2011–2014). Expert Opin Ther Pat.

[12] Cho H (2013). Protein tyrosine phosphatase 1B (PTP1B) and obesity. Vitam Horm.

[13] Zabolotny JM, Kim YB, Welsh LA, Kershaw EE, Neel BG, Kahn BB (2008). Protein-tyrosine phosphatase 1B expression is induced by inflammation in vivo. J Biol Chem.

[14] Ito Y, Banno R, Hagimoto S, Ozawa Y, Arima H, Oiso Y (2012). TNFalpha increases hypothalamic PTP1B activity via the NFkappaB pathway in rat hypothalamic organotypic cultures. Regul Pept.

[15] Lu X, Malumbres R, Shields B, Jiang X, Sarosiek KA, Natkunam Y, Tiganis T, Lossos IS (2008). PTP1B is a negative regulator of interleukin 4-induced STAT6 signaling. Blood.

[16] Zhang J, Wang B, Zhang W, Wei Y, Bian Z, Zhang CY, Li L, Zen K (2013). Protein tyrosine phosphatase 1B deficiency ameliorates murine experimental colitis via the expansion of myeloid-derived suppressor cells. PLoS One.

[17] Traves PG, Pardo V, Pimentel-Santillana M, Gonzalez-Rodriguez A, Mojena M, Rico D, Montenegro Y, Cales C, Martin-Sanz P, Valverde AM, Bosca L (2014). Pivotal role of protein tyrosine phosphatase 1B (PTP1B) in the macrophage response to pro-inflammatory and anti-inflammatory challenge. Cell Death Dis.

[18] Nasimian A, Taheripak G, Gorgani-Firuzjaee S, Sadeghi A, Meshkani R (2013). Protein tyrosine phosphatase 1B (PTP1B) modulates palmitate-induced cytokine production in macrophage cells. Inflamm Res.

[19] Xu H, An H, Hou J, Han C, Wang P, Yu Y, Cao X (2008). Phosphatase PTP1B negatively regulates MyD88- and TRIF-dependent proinflammatory cytokine and type I interferon production in TLR-triggered macrophages. Mol Immunol.

[20] Blasi E, Barluzzi R, Bocchini V, Mazzolla R, Bistoni F (1990). Immortalization of murine microglial cells by a v-raf/v-myc carrying retrovirus. J Neuroimmunol.

[21] Cheepsunthorn P, Radov L, Menzies S, Reid J, Connor JR (2001). Characterization of a novel brain-derived microglial cell line isolated from neonatal rat brain. Glia.

[22] Saura J, Tusell JM, Serratosa J (2003). High-yield isolation of murine microglia by mild trypsinization. Glia.

[23] Liang F, Lee SY, Liang J, Lawrence DS, Zhang ZY (2005). The role of protein-tyrosine phosphatase 1B in integrin signaling. J Biol Chem.

[24] Shen K, Keng YF, Wu L, Guo XL, Lawrence DS, Zhang ZY (2001). Acquisition of a specific and potent PTP1B inhibitor from a novel combinatorial library and screening procedure. J Biol Chem.

[25] Xie L, Lee SY, Andersen JN, Waters S, Shen K, Guo XL, Moller NP, Olefsky JM, Lawrence DS, Zhang ZY (2003). Cellular effects of small molecule PTP1B inhibitors on insulin signaling. Biochemistry.

[26] Ock J, Lee H, Kim S, Lee WH, Choi DK, Park EJ, Kim SH, Kim IK, Suk K (2006). Induction of microglial apoptosis by corticotropin-releasing hormone. J Neurochem.

[27] Kim JH, Jeong JH, Jeon ST, Kim H, Ock J, Suk K, Kim SI, Song KS, Lee WH (2006). Decursin inhibits induction of inflammatory mediators by blocking nuclear factor-kappaB activation in macrophages. Mol Pharmacol.

[28] Jang E, Lee S, Kim JH, Kim JH, Seo JW, Lee WH, Mori K, Nakao K, Suk K (2013). Secreted protein lipocalin-2 promotes microglial M1 polarization. FASEB J.

[29] Jeon H, Kim JH, Kim JH, Lee WH, Lee MS, Suk K (2012). Plasminogen activator inhibitor type 1 regulates microglial motility and phagocytic activity. J Neuroinflammation.

[30] Zhang S, Liu S, Tao R, Wei D, Chen L, Shen W, Yu ZH, Wang L, Jones DR, Dong XC, Zhang ZY (2012). A highly selective and potent PTP-MEG2 inhibitor with therapeutic potential for type 2 diabetes. J Am Chem Soc.

[31] Cho CY, Koo SH, Wang Y, Callaway S, Hedrick S, Mak PA, Orth AP, Peters EC, Saez E, Montminy M (2006). Identification of the tyrosine phosphatase PTP-MEG2 as an antagonist of hepatic insulin signaling. Cell Metab.

[32] Jha MK, Lee S, Park DH, Kook H, Park KG, Lee IK, Suk K (2015). Diverse functional roles of lipocalin-2 in the central nervous system. Neurosci Biobehav Rev.

[33] Jha MK, Suk K (2014). Management of glia-mediated neuroinflammation and related patents. Recent Pat Inflamm Allergy Drug Discov.

[34] Zhang ZY, Lee SY (2003). PTP1B inhibitors as potential therapeutics in the treatment of type 2 diabetes and obesity. Expert Opin Investig Drugs.

[35] Jha MK, Seo M, Kim JH, Kim BG, Cho JY, Suk K (1834). The secretome signature of reactive glial cells and its pathological implications. Biochim Biophys Acta.

[36] Jha MK, Jeon S, Suk K (2012). Glia as a link between neuroinflammation and neuropathic pain. Immune Netw.

[37] Suk K, Ock J (2012). Chemical genetics of neuroinflammation: natural and synthetic compounds as microglial inhibitors. Inflammopharmacology.

[38] Choi DK, Koppula S, Suk K (2011). Inhibitors of microglial neurotoxicity: focus on natural products. Molecules.

[39] Dube N, Tremblay ML (2005). Involvement of the small protein tyrosine phosphatases TC-PTP and PTP1B in signal transduction and diseases: from diabetes, obesity to cell cycle, and cancer. Biochim Biophys Acta.

[40] Bourdeau A, Dube N, Tremblay ML (2005). Cytoplasmic protein tyrosine phosphatases, regulation and function: the roles of PTP1B and TC-PTP. Curr Opin Cell Biol.

[41] Klaman LD, Boss O, Peroni OD, Kim JK, Martino JL, Zabolotny JM, Moghal N, Lubkin M, Kim YB, Sharpe AH (2000). Increased energy expenditure, decreased adiposity, and tissue-specific insulin sensitivity in protein-tyrosine phosphatase 1B-deficient mice. Mol Cell Biol.

[42] Zinker BA, Rondinone CM, Trevillyan JM, Gum RJ, Clampit JE, Waring JF, Xie N, Wilcox D, Jacobson P, Frost L (2002). PTP1B antisense oligonucleotide lowers PTP1B protein, normalizes blood glucose, and improves insulin sensitivity in diabetic mice. Proc Natl Acad Sci U S A.

[43] Krishnan N, Koveal D, Miller DH, Xue B, Akshinthala SD, Kragelj J, Jensen MR, Gauss CM, Page R, Blackledge M (2014). Targeting the disordered C terminus of PTP1B with an allosteric inhibitor. Nat Chem Biol.

[44] Jiang ZX, Zhang ZY (2008). Targeting PTPs with small molecule inhibitors in cancer treatment. Cancer Metastasis Rev.

[45] Pei Z, Liu G, Lubben TH, Szczepankiewicz BG (2004). Inhibition of protein tyrosine phosphatase 1B as a potential treatment of diabetes and obesity. Curr Pharm Des.

[46] Erbe DV, Wang S, Zhang YL, Harding K, Kung L, Tam M, Stolz L, Xing Y, Furey S, Qadri A (2005). Ertiprotafib improves glycemic control and lowers lipids via multiple mechanisms. Mol Pharmacol.

[47] Shrestha S, Bhattarai BR, Cho H, Choi JK, Cho H (2007). PTP1B inhibitor Ertiprotafib is also a potent inhibitor of IkappaB kinase beta (IKK-beta). Bioorg Med Chem Lett.

[48] Bhattarai BR, Ko JH, Shrestha S, Kafle B, Cho H, Kang JH, Cho H (2010). Inhibition of IKK-beta: a new development in the mechanism of the anti-obesity effects of PTP1B inhibitors SA18 and SA32. Bioorg Med Chem Lett.

[49] Lam NT, Covey SD, Lewis JT, Oosman S, Webber T, Hsu EC, Cheung AT, Kieffer TJ (2006). Leptin resistance following over-expression of protein tyrosine phosphatase 1B in liver. J Mol Endocrinol.

[50] Wu Y, Ouyang JP, Wu K, Wang SS, Wen CY, Xia ZY (2005). Rosiglitazone ameliorates abnormal expression and activity of protein tyrosine phosphatase 1B in the skeletal muscle of fat-fed, streptozotocin-treated diabetic rats. Br J Pharmacol.

[51] Dadke SS, Li HC, Kusari AB, Begum N, Kusari J (2000). Elevated expression and activity of protein-tyrosine phosphatase 1B in skeletal muscle of insulin-resistant type II diabetic Goto-Kakizaki rats. Biochem Biophys Res Commun.

[52] Ahmad F, Goldstein BJ (1995). Increased abundance of specific skeletal muscle protein-tyrosine phosphatases in a genetic model of insulin-resistant obesity and diabetes mellitus. Metabolism.

[53] Ozek C, Kanoski SE, Zhang ZY, Grill HJ, Bence KK (2014). Protein-tyrosine phosphatase 1B (PTP1B) is a novel regulator of central brain-derived neurotrophic factor and tropomyosin receptor kinase B (TrkB) signaling. J Biol Chem.

[54] Gonzalez-Rodriguez A, Mas-Gutierrez JA, Mirasierra M, Fernandez-Perez A, Lee YJ, Ko HJ, Kim JK, Romanos E, Carrascosa JM, Ros M (2012). Essential role of protein tyrosine phosphatase 1B in obesity-induced inflammation and peripheral insulin resistance during aging. Aging Cell.

[55] Pike KA, Hutchins AP, Vinette V, Theberge JF, Sabbagh L, Tremblay ML, Miranda-Saavedra D (2014). Protein tyrosine phosphatase 1B is a regulator of the interleukin-10-induced transcriptional program in macrophages. Sci Signal.

[56] Grant L, Shearer KD, Czopek A, Lees EK, Owen C, Agouni A, Workman J, Martin-Granados C, Forrester JV, Wilson HM (2014). Myeloid-cell protein tyrosine phosphatase-1B deficiency in mice protects against high-fat diet and lipopolysaccharide-induced inflammation, hyperinsulinemia, and endotoxemia through an IL-10 STAT3-dependent mechanism. Diabetes.

[57] Arias-Romero LE, Saha S, Villamar-Cruz O, Yip SC, Ethier SP, Zhang ZY, Chernoff J (2009). Activation of Src by protein tyrosine phosphatase 1B Is required for ErbB2 transformation of human breast epithelial cells. Cancer Res.

[58] Matsui H, Harada I, Sawada Y (2012). Src, p130Cas, and mechanotransduction in cancer cells. Genes Cancer.

[59] Roskoski R (2005). Src kinase regulation by phosphorylation and dephosphorylation. Biochem Biophys Res Commun.

[60] Liu ST, Pham H, Pandol SJ, Ptasznik A (2013). Src as the link between inflammation and cancer. Front Physiol.

[61] Byeon SE, Yi YS, Oh J, Yoo BC, Hong S, Cho JY (2012). The role of Src kinase in macrophage-mediated inflammatory responses. Mediators Inflamm.

[62] Lin MT, Lin BR, Chang CC, Chu CY, Su HJ, Chen ST, Jeng YM, Kuo ML (2007). IL-6 induces AGS gastric cancer cell invasion via activation of the c-Src/RhoA/ROCK signaling pathway. Int J Cancer.

[63] Satoh A, Gukovskaya AS, Edderkaoui M, Daghighian MS, Reeve JR, Shimosegawa T, Pandol SJ (2005). Tumor necrosis factor-alpha mediates pancreatitis responses in acinar cells via protein kinase C and proline-rich tyrosine kinase 2. Gastroenterology.

[64] Ramnath RD, Sun J, Bhatia M (2009). Involvement of SRC family kinases in substance P-induced chemokine production in mouse pancreatic acinar cells and its significance in acute pancreatitis. J Pharmacol Exp Ther.

[65] Gong P, Angelini DJ, Yang S, Xia G, Cross AS, Mann D, Bannerman DD, Vogel SN, Goldblum SE (2008). TLR4 signaling is coupled to SRC family kinase activation, tyrosine phosphorylation of zonula adherens proteins, and opening of the paracellular pathway in human lung microvascular endothelia. J Biol Chem.

[66] Liu X, Brodeur SR, Gish G, Songyang Z, Cantley LC, Laudano AP, Pawson T (1993). Regulation of c-Src tyrosine kinase activity by the Src SH2 domain. Oncogene.

[67] Cheng A, Bal GS, Kennedy BP, Tremblay ML (2001). Attenuation of adhesion-dependent signaling and cell spreading in transformed fibroblasts lacking protein tyrosine phosphatase-1B. J Biol Chem.

[68] Arregui CO, Balsamo J, Lilien J (1998). Impaired integrin-mediated adhesion and signaling in fibroblasts expressing a dominant-negative mutant PTP1B. J Cell Biol.

[69] Dadke S, Chernoff J (2003). Protein-tyrosine phosphatase 1B mediates the effects of insulin on the actin cytoskeleton in immortalized fibroblasts. J Biol Chem.

[70] Xin Z, Liu G, Abad-Zapatero C, Pei Z, Szczepankiewicz BG, Li X, Zhang T, Hutchins CW, Hajduk PJ, Ballaron SJ (2003). Identification of a monoacid-based, cell permeable, selective inhibitor of protein tyrosine phosphatase 1B. Bioorg Med Chem Lett.

[71] Zhang ZY (2002). Protein tyrosine phosphatases: structure and function, substrate specificity, and inhibitor development. Annu Rev Pharmacol Toxicol.

[72] Puius YA, Zhao Y, Sullivan M, Lawrence DS, Almo SC, Zhang ZY (1997). Identification of a second aryl phosphate-binding site in protein-tyrosine phosphatase 1B: a paradigm for inhibitor design. Proc Natl Acad Sci U S A.

[73] Sun JP, Fedorov AA, Lee SY, Guo XL, Shen K, Lawrence DS, Almo SC, Zhang ZY (2003). Crystal structure of PTP1B complexed with a potent and selective bidentate inhibitor. J Biol Chem.

[74] Guo XL, Shen K, Wang F, Lawrence DS, Zhang ZY (2002). Probing the molecular basis for potent and selective protein-tyrosine phosphatase 1B inhibition. J Biol Chem.

[75] Taing M, Keng YF, Shen K, Wu L, Lawrence DS, Zhang ZY (1999). Potent and highly selective inhibitors of the protein tyrosine phosphatase 1B. Biochemistry.

[76] Dodd GT, Decherf S, Loh K, Simonds SE, Wiede F, Balland E, Merry TL, Munzberg H, Zhang ZY, Kahn BB (2015). Leptin and insulin act on POMC neurons to promote the browning of white fat. Cell.

[77] Bettaieb A, Bakke J, Nagata N, Matsuo K, Xi Y, Liu S, AbouBechara D, Melhem R, Stanhope K, Cummings B (2013). Protein tyrosine phosphatase 1B regulates pyruvate kinase M2 tyrosine phosphorylation. J Biol Chem.

