# Supplementary figures and images for: A novel role for protein tyrosine phosphatase 1B as a positive regulator of neuroinflammation
Source: J Neuroinflammation. 2016 Apr 19;13:86. doi: 10.1186/s12974-016-0545-3 (PMC4837589; doi:10.1186/s12974-016-0545-3)

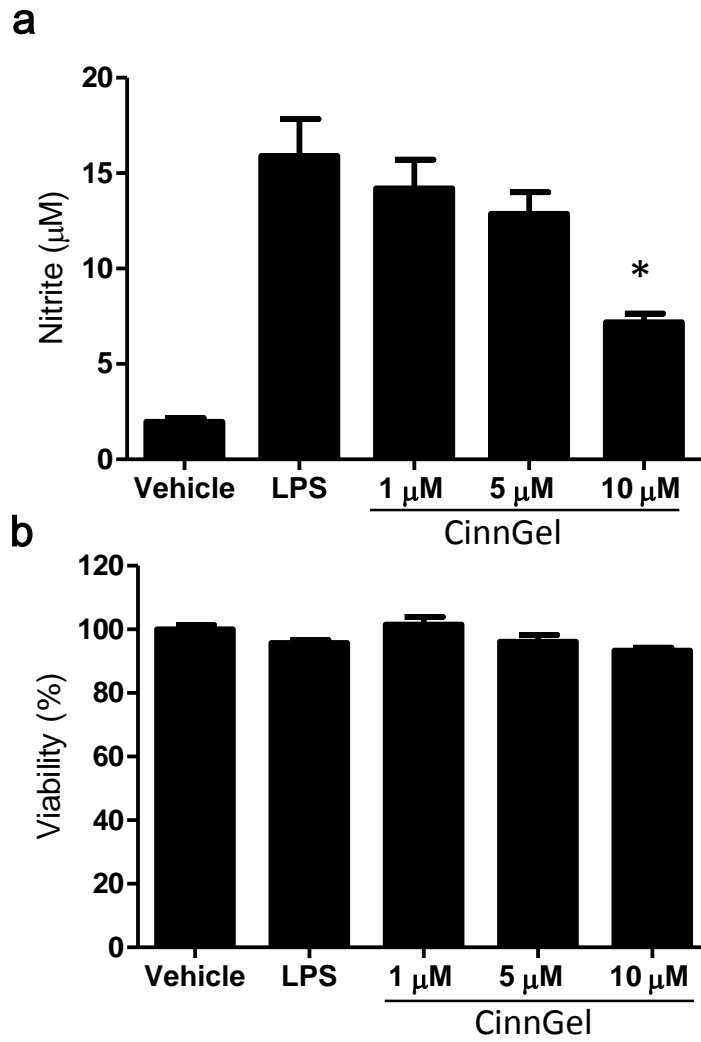

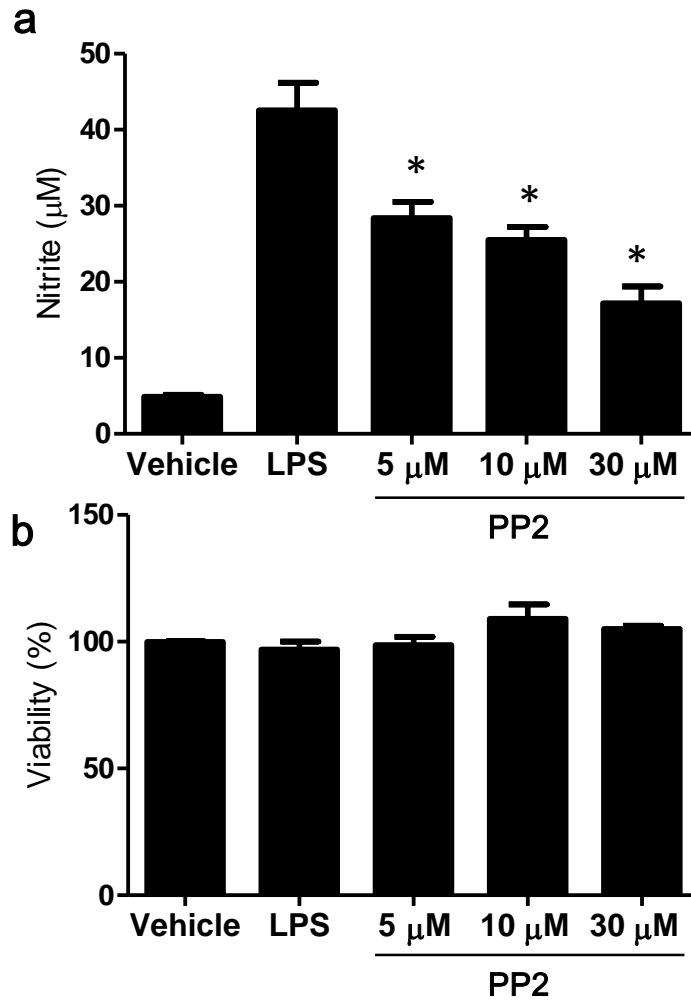

Supplement: Additional file 1: — This contains Figures S1 and S2. Figure S1. The PTP1B inhibitor, CinnGel, suppressed LPS-induced NO production in microglial cells. BV-2 microglial cells were treated with LPS (100 ng/ml) for 24 h after 1 h pretreatment with the indicated concentrations of CinnGel. The nitrite content was measured using the Griess reaction (a) and cytotoxicity of PTP1Bi was assessed by the MTT assay (b). The data were expressed as the mean ± SEM (n = 3). *p < 0.05 versus LPS only, one-way ANOVA with Tukey’s multiple comparison test. Figure S2. The Src inhibitor, PP2, suppressed LPS-induced NO production in microglial cells. BV-2 microglial cells were treated with LPS (100 ng/ml) for 24 h after 1 h pretreatment with the indicated concentrations of PP2. The nitrite content was measured using the Griess reaction (a) and cytotoxicity of PTP1Bi was assessed by the MTT assay (b). The data were expressed as the mean ± SEM (n = 3). *p < 0.05 versus LPS only, one-way ANOVA with Tukey’s multiple comparison test. (PDF 174 kb) [file 12974_2016_545_MOESM1_ESM.pdf]
